# Existing workloads among managers and healthcare workers during the COVID-19 pandemic: Meanings in the Amazon context

**DOI:** 10.1371/journal.pone.0292541

**Published:** 2023-10-05

**Authors:** Wagner Ferreira Monteiro, Kássia Janara Veras Lima, Darlisom Sousa Ferreira, Lucas Lorran Costa de Andrade, Flávia Regina Souza Ramos

**Affiliations:** 1 Department of Graduate Studies in Tropical Medicine, Tropical Medicine Foundation Dr. Heitor Vieira Dourado, Manaus, Amazonas, Brazil; 2 Department of Nursing, School of Health Sciences, Amazonas State University, Manaus, Amazonas, Brazil; 3 Graduate Program in Public Health Nursing, State University of Amazonas, Manaus, Amazonas, Brazil; 4 Nursing Graduate Program, Federal University of Santa Catarina, Florianópolis, Santa Catarina, Brazil; Tehran University of Medical Sciences, ISLAMIC REPUBLIC OF IRAN

## Abstract

**Introduction:**

The global COVID-19 pandemic has increased the concern and risks of health professionals exposed by acting on the front lines in combating and controlling the spread of the virus. This study aims at analyzing the workloads and their implications for the activities carried out by managers and health workers in the face of the COVID-19 pandemic in Manaus, Amazonas, Brazil.

**Method:**

This is a qualitative study, of the case study type, that integrates a macro research that deals with the experiences built during the confrontation of the pandemic of COVID-19 in the capital of the state of Amazonas, Brazil, the epicenter of the pandemic in the country. Data production resorted to documentary analysis and semi-structured interviews with 56 managers or professionals from the Manaus Health Care Network. The analytical process was supported by the resources of the Atlas.ti 8.0 software and the precepts of Thematic Networks analysis.

**Results:**

The thematic network “workloads” brought together three topics related to the experience of psychological, physiological and biological loads. Psychological workloads were strongly present in the work routine, being referred to in a more significant way. The sources that increase them are strongly related to work stress, dealing with suffering and death and excess demand on the service. Physiological loads were related to excessive working hours, staff shortages and activity overload. “Biological burdens” include exposure to the SARS-CoV-2 virus, constant contact with infected individuals, and individual protection as key elements of this set.

**Conclusion:**

The study showed that both managers and workers have work processes and environments with conditions that tend to generate workloads that harm their health and safety, as well as institutions and patient care. Therefore, there is a need for more effective organizational actions in workers’ health surveillance, disease prevention, adequate working conditions, reducing workloads and promoting more resolute and less stressful work environments.

## Introduction

The emergence of a severe acute respiratory syndrome coronavirus (SARS-CoV-2) in China in late 2019 led to a global pandemic, drastically affecting every country in the world [1,2]. While official data express the magnitude and severity of the pandemic in terms of numbers of cases and deaths [3], health workers and managers have faced and continue to face unprecedented professional challenges [4].

This group of professionals works under extreme pressure, exposed to high stress levels, in long shifts, excessive hour loads and, sometimes, without adequate training or personal protective equipment [5,6]. In addition to that, they face unprecedented situations, such as allocating scarce resources to equally needy patients, providing assistance with limited resources and lack of adequate therapy, which exerts a direct impact on work-related burdens during disease outbreaks [7,8].

With the rapid spread of the disease, the concern with health professionals exposed for working on the front line in combating and controlling propagation of the virus increases [9], in order to ensure the safety of those who care for infected people [10].

Based on recent evidence, the World Health Organization (WHO) has published guidelines for infection control and contamination prevention in health care services for infectious diseases such as COVID-19 [11]. However, as the pandemic spread globally, different physical and psychological problems and repercussions were reported affecting health workers and managers, such as concerns around the risks of contracting the disease or transmitting it to family members, feelings of inability when faced with seriously-ill patients and intense and exhausting working hours [9].

The health work process is developed under conditions that generate workloads, which act either directly or indirectly on workers’ health and on development of patient care [12]. It is clear that working conditions and human factors affect continuous provision of care, its results and patient safety [13].

Workloads are characterized as elements present in the work process that, when dynamically interacting with each other and with the workers, can be responsible for new adaptation processes and, as a consequence, with wear out in workers, which is defined as loss of physical and psychological capacity of these professionals. The physical, chemical, biological and mechanical loads are characterized as external materiality loads. In turn, the physiological and psychological loads are characterized as internal materiality loads [14]. They are determined by factors that are oftentimes not clearly identified by the professionals themselves. Knowing the elements that contribute to the increase and reduction of workloads helps to strengthen the positive aspects of work and to minimize the negative ones [15].

In this sense, the study aims at analyzing the workloads and their implications for the activities carried out by managers and health workers in the face of the COVID-19 pandemic in Manaus, Amazonas, Brazil.

### Workloads in the face of the pandemic

The rapid transmission of COVID-19 has had a profound impact on the routines of thousands of people around the world. This situation has required the mobilization of a wide range of health workers to respond promptly to the growing care needs in the various health services [16].

There is substantial evidence of high exposure and contamination of healthcare workers by COVID-19. Studies identify an association between longer working hours, inadequate hand hygiene, and risk of infection [17], it is estimated that 4% to 12% of confirmed cases are health professionals. The data reveal the dimensions of the problem, with a dynamic scenario, especially because these workers experience various workloads and consequences in their work and health, beyond the COVID-19 itself but arising from it [18].

Health professionals face precarious working conditions on a daily basis, in an environment marked by lack of safety, inadequate infrastructure, and risks, favoring high levels of occupational exhaustion, physical and mental illness, and poor quality of care. These situations are more common for front-line personnel fighting COVID-19 due to long working hours, patient health concerns, and scarcity of personal protective equipment, especially in countries with limited resources [19,20].

Given this situation, research on workloads [14] can help understand the exposure dynamics of health care workers from the first months of the pandemic. Although loads and stresses do not mean illness or have a linear relationship with it, they are important elements in the prevention of illnesses and promotion of workers’ health.

## Method

This is a qualitative study of the case study type, which integrates a macro-research dealing with the experiences built in the course of facing the COVID-19 pandemic, in the scenario under study. Suitability of the study design is due to the fact that it defines a complex phenomenon (response to the pandemic) in a specific context (municipality), in a combination of different analysis units (different types of services and management levels) and sources of evidence (interviews with different informants and documents).

Developed in Manaus, the capital city of Amazonas with 2,219,580 inhabitants and representing 52.75% of the state’s population, 13.01% of the North Region and 1.04% of Brazil, being the seventh most populous capital [21]. In order to fight against COVID-19, the Health Care Network (HCN) was restructured with priority entry points: Basic Health Units (BHUs), Urgency Services (US), First Aid Units (FAUs) and Emergency Services (ER). Admissions to an Intensive Care Unit (ICU) or clinical unit occur through medical supervision, transportation by the Supervised Emergency Transfer System (*Sistema Supervisionado de Transferência de Emergência*, SISTER) and by the Mobile Emergency Care Service (*Serviço de Atendimento Móvel de Urgência*, SAMU) [22,23].

A total of 56 participants took part in the study, 23 of which were managers and 33 health workers linked to the services involved in dealing with COVID-19, meeting the minimum representativeness of the different health services and using as an inclusion criterion having worked in that service for at least 01 month. This period of time was considered sufficient by the data saturation criterion, when non-emergence of new explanations or descriptions about the phenomenon under study was evidenced [24]. Data production took place in the second half of 2020, resorting to semistructured interviews and documentary analysis.

The interviews took place at the workplace and were conducted by the main researcher or a duly trained employee. A previous script was used, consisting of 11 questions that addressed the socio-professional profile, as well as 8 guiding questions focused on experiences during the pandemic. These questions explored topics such as the perception of coping with COVID-19, the impact on health activities, the adaptation of teams to the new guidelines and work structures, the influence of the work environment in this context—and, fundamentally, the different workloads experienced. Each interview lasted an average of 45 minutes, was audio-recorded then fully transcribed using the Google Docs tool. This approach ensured the fidelity and accuracy of the information recorded.

The diverse information transcribed was then imported into the Atlas.ti 8.0 software (*Qualitative Research and Solutions*) [25] for data analysis; its understanding was through the precepts of the analysis of Thematic Networks and its interpretation was based on the conceptual matrix adopted. Application of thematic networks is a way of organizing the analysis of qualitative data, seeking to discover the topics highlighted in a text, aiming to facilitate structuring and representation of these topics, exploring understanding of an issue or the meaning of a conception, which start from breaking down the text into explicit grounds and implicit meanings [26].

The lead researcher independently carried out inductive coding of the transcripts to identify and compile a preliminary list of codes. To ensure the reliability of the process, a team of four researchers analyzed and discussed these codes, arriving at a definitive set that served as a framework for analysis, the interpretation of preliminary results, and the identification of key patterns. Using these insights, a second round of coding was carried out, with the aim of identifying patterns in the initial coding and selecting representative quotes for each theme. These themes were then grouped into three broad categories, which gave rise to the Workload Thematic Network. Other thematic networks generated in the analysis, related to the organization of work, work environments and experiences of fear in the fight against COVID-19 have been explored by the authors in other manuscripts.

The documentary research covered freely accessible documents on websites of national, state and municipal bodies, organized in a specific Google drive, totaling 265 documents. They were systematized in regulatory, protocol and information system data and added to the analysis to organize and deepen the interpretations.

All the ethical precepts related to research involving human beings and to the National Health Council constant devices were respected. Permission was also obtained from the State and Municipal Health Departments to carry out the research, and approval was obtained from the Research Ethics Committee of the State University of Amazonas. All the participants gave their consent by signing the Free and Informed Consent Form, guaranteeing data confidentiality and anonymity by coding the participants. In the excerpts, the statements made by the study participants are identified with the letters M (Manager) and HW (Health Worker), followed by a number representing the order in which they were interviewed.

## Results

The study involved 56 participants aged from 24 and 60 years old, predominantly female (64.3%), and with more than 5 years of professional experience (80.3%). The participants’ socio-occupational profile is presented in Table 1.

**Table 1:** Socio-occupational profile of the managers and health workers from the Health Care Network devoted to the fight against COVID-19, Manaus, AM, 2021.

Variables	Definition	Number of Managers	Number of Health Workers	Total
**Gender**	Female	12	24	36
	Male	11	9	20
**Age**	20–40 years old	07	17	24
	41–50 years old	13	14	27
	51–60 years old	03	02	05
	60+ years old	--	--	--
**Education**	High School	--	08	08
	Graduation	01	09	10
	Specialization	15	15	30
	Master’s degree	06	01	07
	PhD	01	--	01
**Job experience**	1–5 years	--	11	11
	5+ years	23	22	45
**Time working in the current institution**	1 year	04	10	14
	1–5 years	06	08	14
	5+ years	13	15	28
**Type of contract**	Public tender	15	17	32
	Commissioned	05	--	05
	Temporary	--	02	02
	Outsourced	03	10	13
	Scholarship holder	--	04	04
**Performance level**	Primary Care	11	19	30
	Secondary care	07	07	14
	Tertiary care	05	07	12
**Workload (weekly)**	Less than 40 hours	--	--	--
	40 hours	15	16	31
	More than 40 hours	08	17	25
**Other employment contract**	Yes	08	18	26
	No	15	15	30

Source: Research database.

**Fig 1:**
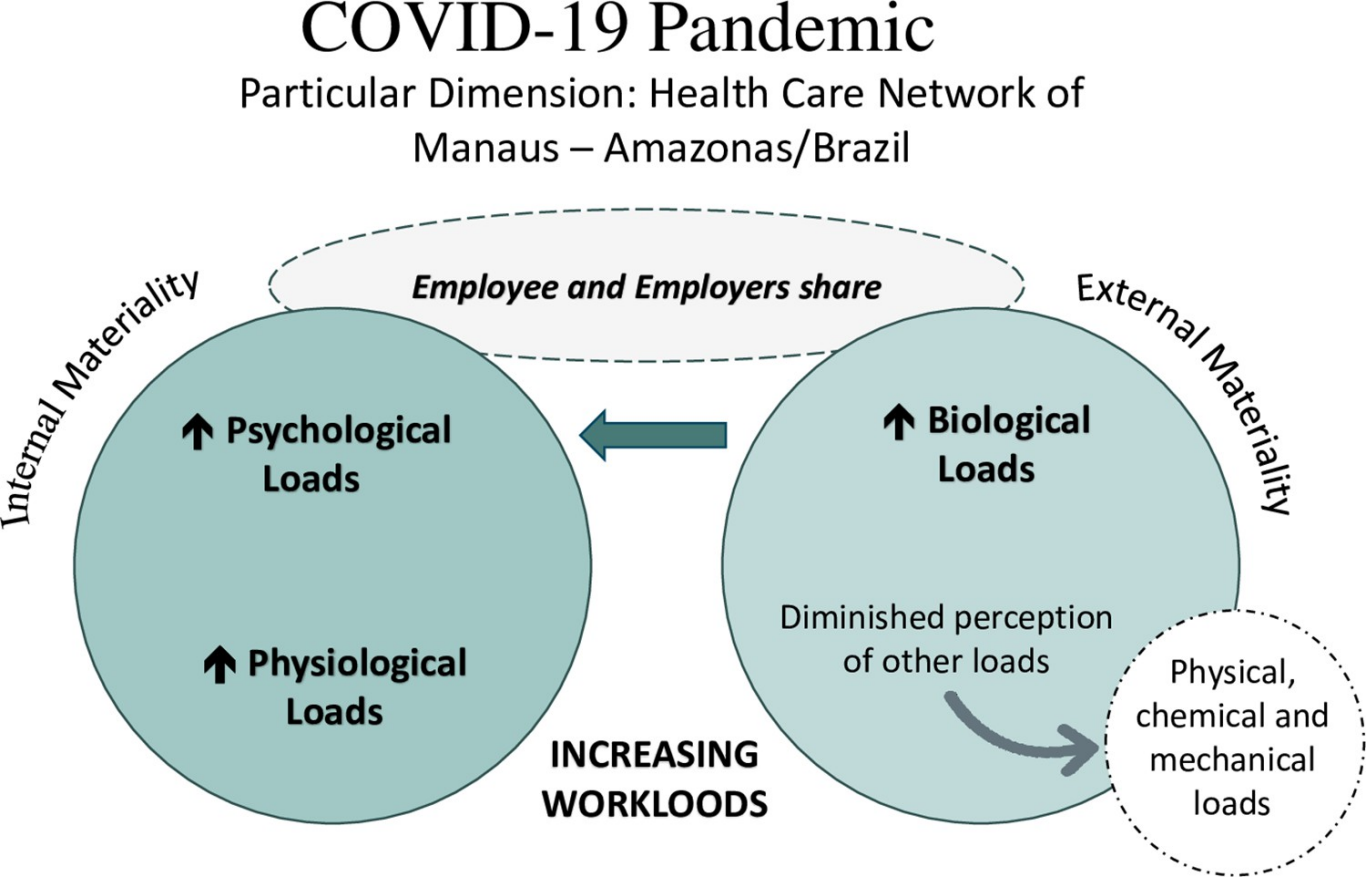
Procedural model of the workloads referred by managers and health workers in the work process in the COVID-19 pandemic in Manaus, AM, 2021. Source. Prepared by the authors based on the theoretical-methodological approach of Laurell & Noriega, 1989 with resources from the Microsoft 365® PowerPoint® program.

The “workloads” thematic network gathered three topics related to the experience of psychological, physiological and biological loads, as synthesized in Fig 1, based on Laurell & Noriega’s theoretical-methodological approach (1989). It is worth noting that the psychological load, which corresponds to an internal materiality load, was the element mentioned with the most emphasis by the interviewees. In contrast to this aspect, the chemical, physical and mechanical external materiality loads did not emerge in the interviews.

Psychological workloads are strongly present in the interviewees’ work routine, being the most significant. In addition to that, the sources that increase them are strongly related to stress at work, to dealing with suffering and death, and to excess demand in the service.

The impact of the precarious conditions, especially at critical moments, emotionally marked the workers, to the point that the stress culminated in despair and in absence of any positive expectation when starting each work shift. The interviewees stated the following specifically regarding this topic:

Constant stress. I arrived expecting the worst, I came here and the nurses were all but overloaded and the emergency room was overcrowded. Sometimes with a patient aged 30 something saturating at 70% maximum O_2_ level, with the need to be intubated and there was no mechanical ventilator for him (HW 12).

[…] we had a death case inside the ambulance, at the hospital door, but the hospital took its time to receive him, because there was no place to do so. We even had the case of one of our employees who died […] being treated at the hospital and having to share the oxygen with another patient who was next door, because there was no oxygen nozzle, there weren’t enough oxygen outlets. Then, that stressed us all a lot (M 4).

It was so exhausting, it got to be desperate, because you had to deal with the fear of the patient and of your colleague, our employee. The employee was there, assisting in the best possible way, but also afraid of getting sick, afraid to take it to his family, afraid of getting his family sick, and really, especially during April and May, every week two to three employees were distanced due to COVID. Thank God we didn’t have any death; one of our employees only, she had to be hospitalized, but for a short time. For a long time it was a very big mental wear out (M 16).

The already expected wear out of living with suffering and death reaches new and unexpected dimensions when exacerbated by constant pressures, uncertainties in the face of the unknown, frustration and impotence. All that can become unbearable for some people and excessive for all.

Watching a person suffer and lacking the specific structure to be provided, to try and alleviate that suffering, it’s very complicated. I got home very stressed due to the mere fact of dealing with a new agent […] that you don’t know much about, it’s stressful (HW 24).

I think that was the heaviest, we were under huge pressure, not only from the need to give a result to society, with people dying there every day, more than 150 burials in Manaus, pressure from the media, from everything that was happening, the whole world watching us. There was pressure from our top management, the mayor, for us to make everything happen in the best possible way, with the urgency required (M 3).

It was really frustrating, because I thought that I was prepared and that I had prepared my colleagues, but when we started to deal with the cases, we saw very significant particularities for the condition of saving the patients’ lives. And we learned from our mistakes, we turned these frustrations into experiences and, from there, we managed to better deal with these patients and with the emotional issue, because it was sad, arriving here and having three or four deaths in a few hours. That devalued our job, because we had done everything we could, we studied, applied the treatment and then, when the time comes, everything is wrong. It’s really frustrating (M 11).

Seen as necessary for providing care effectively, the different actions developed in order to solve the emerging demands of the work context in the pandemic appear as a psychological load. Most of these actions are related to the services but, at some point, the high demands for care and due to illness and death of employees overwhelm others still active, both physically and mentally. It is an issue that has repercussions among the work groups with emotional exhaustion, according to the interviews.

We had a lot of problems with employees, increasing the hour load and overloading teamwork. Many employees ended up contaminated and being distanced, overloading those who stayed here. Then, at some moment we had more than 100 employees distanced due to COVID-19; it was the most complicated and complex time we went through (M 7).

I had a shift, I will always remember that shift, it was on a Saturday, the other technicians had already been distanced and I only had two technicians left; it was me and the two technicians on a shift that had five deaths. I couldn’t take it anymore in this shift, physically I couldn’t take it anymore, I couldn’t take it emotionally anymore, it was very exhausting (HW 5).

Wear out was intense, because there was both emotional and physical overload. The mental part was heavy, because we lived in that war environment, everyone “covered” here, it was a really charged environment. So, the psychological part was really affected and the physical part too, because many contaminated people were distanced (M 8).

Regarding exposure to the physiological loads, according to the participants, the biggest problems are due to the excessive working hours, to the deficit of employees and to the overload of activities. These are almost always the result of disordered processes and environments.

Work practically tripled […] so the workday increased so much that the staff was not enough, relocated and adaptation were necessary. […] sometimes we doubled the shift. Then we had to go through this adaptation, many employees got sick at the beginning, due to this contact and for not knowing how to deal with the disease itself. There was the psychological issue that affected many employees, afraid, not wanting to work… they got sick or afraid of the disease (HW 21).

At certain moments we had more than 200 employees distanced and others, like me, at a not too young age, with diabetes problems, belonging to the risk group. We took the initiative to distance them, even if they were not sick, nearly 180 who were actually distanced […]. And we couldn’t, we even had the ambulance at the time, but we didn’t have anyone to man it (M 4).

Particularly in the health work process, the manifestation of physiological loads occurs in the workers’ interaction with their work object (the patient being cared for), generating wearing processes, until exhaustion and the onset of a series of signs and symptoms that are often nonspecific and difficult to relate to work. There was a strong overlap between the physiological and the psychological loads, to the point of recognizing an inescapable situation of reverberation and overlapping workloads, with impacts still to be understood in the long term.

Work was excessive, I worked a whole lot more than 12 hours, I had my time to enter the pink room, but no time to go out. They changed shift after shift, because the colleague couldn’t leave. […] I had an anxiety crisis, because you’re under that tension all the time (HW 29).

The workload was very intense because it all came together, it wasn’t only demand or stress, it was all together in fact, I suffered due to that, mainly at night […] my sleep and wake cycle was completely altered and it took me months to understand (HW 30).

Topic 3, “experiencing biological loads” included exposure to the SARS-CoV-2 virus, constant contact with infected individuals, and personal protection as main elements in this set.

We used to say:—let’s go to the "COVID ward", there for isolation. So, I was already afraid to go, not only because of contamination, but knowing that because of the viral overload level, it were more complicated cases that came in… and we began to see ourselves with that pressure, that we had to respond to that, urgency and even emergency (HW 25).

It was complicated at the beginning because we didn’t know the virus, I was afraid of going to work. When I was here I didn’t drink water because I was afraid to take off my mask and get contaminated, I wore gloves with alcohol. If you look at it from a before-the-pandemic perspective, it would be really paranoid. Before having lunch I washed myself from head to toes, used the trunk of the car to store my things and took off all my clothes before entering the house. Then I went straight to the shower. I was afraid, I thought I had the coronavirus every day (HW 26).

And the concern I had, we had to visit the units […] I even had a flu, but it was not confirmed as COVID, and at that moment I was thinking about the workers, because they were very vulnerable there […] I think I was at risk, but nothing equal to the risk of contagion in the health workers who were in the field (M 22).

In the set of risks, health problems and aggravated loads, managers and workers highlighted the biological factor, inherent in a pandemic, as well as its dynamics in generating interference in the work overload, in their private lives and harms to their mental health. In addition to the direct standard precautions and PPE use on individual protection, there is a secondary effect, not of less importance and constituting a protective factor against wear out and illness: the sense of bonding and support when workers perceive themselves as a focus of attention and care by the management. Commitment to ensure these materials in adequate numbers and quality promotes a positive bond between managers and workers, facilitating the responses to the needs imposed by the pandemic moment.

The PPE issue—as much as you bring all the knowledge, the organization of the work process, was unanimous in all the virtual meetings or even in the monitoring and follow-up of the teams, in the units, in the services: there should never be PPE shortage, it is for the sake of the professionals’ safety. So it was a main point. I had employee who felt much safer because he knew from the beginning that he had access to personal protective equipment (M 17).

There was mainly the PPE issue, visors were distributed, thicker lab coats; it was the director who arranged it and the issue of reducing the number of people in the sector as much as possible. The instruments were gradually adapted. They got the N95 mask […] some things were very difficult to adapt to, for example this N95, we rejected a little because it is very difficult to breathe and then we ended up adapting, because it was necessary for our protection (HW 14).

The unit’s manager was always ahead of the pandemic. He didn’t miss a minute, he always worked as best he could. We were never out of PPE, never. Some places didn’t have masks or gloves. Here we always had everything, inside the office, gel alcohol. It never failed, so starting from the unit’s manager we were more relaxed. Another thing on the part of the manager was that when an employee was absent because of positive COVID, he provided assistance. I think it was the difference (HW 26).

## Discussion

With regard to the socio-occupational profile of the managers and health workers in the scenario researched, the results were similar to those described in a study carried out in Brazil [27] and internationally [28], with predominance of the female workforce and at least 40 working hours per week.

The analysis of the workloads arising from facing a pandemic highlights the relationship between working conditions and organizational factors to which managers and workers are exposed, despite the fact that this relationship has a historical-social process, according to Laurell & Noriega’s perspective [14], that is, as dynamic and socially determined components. Thus, certain aspects present in the environment and in the process potentiated the workloads more intensely.

From the results obtained, it is observed that in the scenario under study, managers and workers are exposed to psychological, physiological and biological loads, listing a set of elements that, in their perception, increase these loads. For the most part, these elements are in line with research studies that investigate similar situations, where the unexpected was part of these professionals’ routine, increasing psychological wear out and predisposing them to developing health problems [29,30].

However, some peculiarities about the workloads of this study should be considered. It was noticed that the professionals did not identify elements related to the chemical, physical and mechanical external materiality loads. This can be associated with their low understanding about the effects of these loads on their body. The elements involving the workloads are not always clearly perceived by the professionals [15]. The pandemic moment is certainly drawing the professionals’ attention to other loads.

Therefore, it is possible to attribute a central position to the relationship between the work process and the loads in the specific analysis herein developed; however, it is worth noting that the psychological loads were more emphatically present in the participants’ testimonies, evidenced by the fear of being infected, exponentiated by the responsibility to contribute to facing the biggest public health challenge in decades, which explains the conditions of mental disorders [31], anxiety, anguish, insomnia and depression among managers and health workers [32–34]. Stress conditions related to concern for family members and wear out due to excess demand in the service were also mentioned [35].

It was therefore verified that, based on a central relationship made evident (exposure to the new coronavirus), a series of loads of another nature was unfolded or reinforced. It should be noted that they interact with each other and with the internal and external elements of the work process, coated with dynamism [14] and generating wear out processes, especially due to COVID-19 itself and to the psycho-emotional problems [35]. Certainly, part of these loads is more or less present in the professionals’ routine, regardless of the pandemic, but they are potentiated by the tension inherent to this atypical moment, directly contributing to the emergence of destructive processes in the work environment, causing dissatisfaction in workers, compromising quality of life and contributing to their illness [36].

The literature reveals that, in addition to the excess demand in the service, the factors that most potentiate the loads are related to the precariousness of the work environment and to the deficit of employees [15], legitimizing the data exposed in the first and second topics, identifying that the working conditions and the everyday needs are key components in increasing workload and overload of activities.

The physiological workloads were the result of problems such as overload of activities and insufficient number of employees, which culminate in processes of accentuated physical wear out, materialized in physical and mental exhaustion among the main problems that led to shortage of professionals [37]. Absenteeism influences care quality, as it reduces the number of workers, generating an unhealthy environment for those who continue to work [38], corroborating the findings of this study.

Finally, with the lowest intensity among the topics, the biological loads are evidenced by exposure to the virus and contact with infected individuals. As they have materiality external to the worker’s body, these elements are easily identified in the work environment; however, the low intensity related to this load can be due to the pandemic itself, categorizing them as psychological loads [31].

Adopting protective measures for workers’ safety and health is an important strategy to cope with the biological risks existing in the health services. It is responsibility of the institutions to ensure permanent access of the employees to PPE, in consonance with the reality of the services, contemplating the reduction and propagation of communicable diseases and promoting the professionals’ safety, health and well-being [39,40].

Implementing protective measures for the safety of these professionals involves prioritizing investments in measures aimed at infection control and at availability of equipment for individual and collective protection, in addition to valuing work, mainly by government entities, providing benefits on the physical and mental health of managers and workers [41].

The findings of this study strengthen the scientific production on workloads, identifying activities present in the work environments of the health services that can cause physical and psychological stress on workers’ health. The activities evaluated highlight the social relationships experienced by the managers and workers, such as stress, excessive working hours and excess of activities. They also identified a condition related to reducing the workload through the use of individual protection.

It is worth noting that understanding the activities performed at work, as well as appropriate work environments, can improve the quality of patient care and reduce the professionals’ workload, increasing team and patient satisfaction [30].

Understanding the dynamics of the workloads and how they materialized in the pandemic context is crucial to developing and implementing actions that guarantee workers’ safety and well-being, ensuring adequate working conditions and providing not only technical but also psychological support, in the face of the challenges inherent to coping with a pandemic.

The finding that there is a strong feeling of commitment to the patients and of significant dissatisfaction with the working conditions reveals a paradox with which managers and workers live in their everyday lives and which can be an important element of workloads.

## Considerations

The study evidenced that both managers and workers have processes and work environments with conditions that tend to generate workloads, and that these loads can affect their health. Workloads of internal and external materiality were evidenced in the statements, with the psychological workload standing out, responsible for more wear out among the professionals, manifested in altered physical and mental processes, such as stress. Other loads also affect the entire collective of professionals, interacting between each other and with the workers’ body, which can increase physical, and mainly psychological, wear out.

It was concluded that the workloads impair managers’ and workers’ health, safety of the institutions and the assistance provided to the patients. Therefore, there is a need for more effective organizational actions in the surveillance of workers’ health, disease prevention, adequate working conditions, reduction of sources of psychological illness and promotion of more resolute and less exhausting work environments.
